# Testicular expression of the *Lin28*/*let-7* system: Hormonal regulation and changes during postnatal maturation and after manipulations of puberty

**DOI:** 10.1038/srep15683

**Published:** 2015-10-23

**Authors:** S. Sangiao-Alvarellos, M. Manfredi-Lozano, F. Ruiz-Pino, S. León, C. Morales, F. Cordido, F. Gaytán, L. Pinilla, M. Tena-Sempere

**Affiliations:** 1 Department of Cell Biology, Physiology and Immunology, University of Córdoba; CIBER Fisiopatología de la Obesidad y Nutrición, Instituto de Salud Carlos III; and Instituto Maimónides de Investigación Biomédica (IMIBIC)/Hospital Universitario Reina Sofía, 14004 Córdoba, Spain; 2 Department of Pathology, University of Córdoba, 14004 Córdoba, Spain; 3 Department of Medicine, School of Health Science, University of A Coruña, and Instituto de Investigación Biomédica de A Coruña (INIBIC), 15006, A Coruña, Spain; 4 FiDiPro Program, Department of Physiology, University of Turku, Kiinamyllynkatu 10, FIN-20520 Turku, Finland

## Abstract

The Lin28/let-7 system, which includes the RNA-binding proteins, Lin28a/Lin28b, and let-7 miRNAs, has emerged as putative regulator of puberty and male gametogenesis; yet, its expression pattern and regulation in postnatal testis remain ill defined. We report herein expression profiles of Lin28 and let-7 members, and related mir-145 and mir-132, in rat testis during postnatal maturation and in models of altered puberty and hormonal deregulation. Neonatal expression of Lin28a and Lin28b was low and rose markedly during the infantile period; yet, expression patterns diverged thereafter, with persistently elevated levels only for Lin28b, which peaked at puberty. Let-7a, let-7b, mir-132 and mir-145 showed profiles opposite to Lin28b. In fact, let-7b and mir-145 were abundant in pachytene spermatocytes, but absent in elongating spermatids, where high expression of Lin28b was previously reported. Perturbation of puberty by neonatal estrogenization reverted the Lin28/let-7 expression ratio; expression changes were also detected in other models of delayed puberty, due to early photoperiod or nutritional manipulations. In addition, hypophysectomy or growth hormone (GH) deficiency revealed regulation of this system by gonadotropins and GH. Our data document the expression profiles of the Lin28/let-7 system in rat testis along postnatal/pubertal maturation, and their perturbation in models of pubertal and hormonal manipulation.

The heterochronic gene, *Lin28*, was first identified in the nematode, *Caenorhabditis elegans*, where it displays tissue- and stage-specific patterns of expression^1^. In mammals, two *Lin28* related genes, named *Lin28a* (also termed *Lin28*) and *Lin28b*, have been described^2^,^3^,^4^. *Lin28* paralogs are highly conserved across evolution^5^ and they act as post-transcriptional regulators by virtue of their RNA-binding activity. In addition to various coding mRNAs, Lin28 proteins have been shown to bind to the terminal loops of precursors of the *let-7* family of microRNAs (miRNAs), blocking their processing into mature miRNAs^6^. MiRNAs are small, non-coding RNAs that control the expression of a wide range of protein-coding genes. MiRNAs function mainly post-transcriptionally by interacting with specific seed regions at the 3’-UTR of target genes, thereby affecting the stability or translation of the corresponding mRNAs^7^,^8^. Recent evidence has unveiled the central position of Lin28a/Lin28b within a key regulatory network involving also c-Myc and *let-7*^9^,^10^,^11^,^12^; *let-7* miRNAs being highly conserved across phyla, and widely and abundantly expressed in numerous species^13^. The complexity of this regulatory system is illustrated by the fact that Lin28a/Lin28b redundantly represses the synthesis of mature *let-7* miRNAs, which in turn are able to suppress Lin28 levels, therefore creating a double-negative feedback loop. In addition, Lin28a/Lin28b expression is indirectly supressed by *mir-145,* which causes inhibition of expression of c-Myc^14^,^15^, a transcriptional activator of *Lin28a/Lin28b*^11^,^12^. In addition, *mir-132* (as well as *mir-9*) has been predicted as putative miRNA repressor of *Lin28* by the use of bioinformatic algorithms^16^.

In the last few years, the Lin28*/let-7* system has been proposed as an essential regulator of several endocrine systems. Human genome-wide association studies (GWAS) have been reported that *LIN28B*, among other *loci*, might participate in the control of height^17^, growth^18^,^19^ and the timing of puberty^20^,^21^,^22^,^23^. Transgenic mice overexpressing *Lin28a* presented increased body size and delayed onset of puberty^24^, and the overexpression of *Lin28a* and *Lin28b* promoted an insulin-sensitized state, in direct contrast to overexpression of *let-7*, which resulted in insulin resistance and impaired glucose tolerance^25^. In a recent paper, Shinoda *et al.* demonstrated that conditional *Lin28a* or *Lin28b* deletion during fetal period, but not during the neonatal period or adulthood, resulted in growth defects and aberrations in glucose metabolism. Moreover, they observed that *Lin28b* regulates growth in mice in a gender-specific manner, since *Lin28b* KO males, but not females, showed post-natal dwarfism^26^.

Puberty is a crucial developmental event in sexual and somatic maturation, and hence in the life-cycle of any individual^27^,^28^,^29^. In males, the process of spermatogenesis is essential for normal reproduction; the first spermatogenic wave is completed at puberty. This is a complex event, regulated by a large number of factors, including miRNAs^30^,^31^,^32^. Accordingly, up-stream regulators of miRNA synthesis, like *Lin28a*, have been studied as potential modulators of spermatogenesis^33^. *Lin28a* regulates primordial germ cell development in mice^34^ and recently it has been suggested that *Lin28a* is involved in maintaining the identity of adult spermatogonial stem cells (SSCs) in the monkey and human testis^35^. Its deletion results in a reduced germ cell pool in mouse embryos, whereas over-expression causes increased germ cell number^24^,^34^. Elimination of *Lin28a* in mice compromises the size of the germ cell pool in mice, both in males and females, by affecting primordial germ cell proliferation during embryogenesis, thereby leading to reduced fertility in adults^36^. Moreover, *Lin28a* KO males showed altered levels of FSH and testosterone^36^. In the same way, the embryonic over-expression of *let-7* provoked a reduction of the germ cell pool^36^. Using a conditional knockout of *Lin28a* in adult germ line stem cells, Chakraborty *et al.* showed that the loss of *Lin28a* reduced testis weight, sperm number and impaired spermatogonial cell proliferation without compromising their differentiation capacity^37^.

However, in spite of these recent studies, relatively little is known about the pattern of expression and regulation of the Lin28*/let-7* system in the developing and mature male gonad. We report here the expression profiles of key components of the above regulatory pathway, and several associated factors, such as *mir-145* and *mir-132*, in the rat testis during male postnatal/pubertal maturation. In addition, we characterize changes in the expression patterns of these elements in various models of altered puberty and study the role of different hormonal axes, including the gonadotropic system, in the regulation of their testicular expression.

## Methods

### Animals and drugs

All experiments and animal protocols included in this study were reviewed and approved by the Ethics Committee of the University of Córdoba, and were conducted in accordance with EU Normative for the use and care of experimental animals. Male Wistar rats of different ages were used in this study, except in the studies of GH deficiency, where Lewis rats were used. The day the litters were born was considered day 1 of age. Animals were housed in a temperature-controlled room with a standard 14-h/10-h light-dark cycle (lights from 05:00 to 19:00 h), unless otherwise stated (see photoperiod manipulation). Animals were weaned at postnatal day 21 (PND-21) and were provided with *ad libitum* access to water. Hypophysectomized (HPX) rats were purchased from Charles River (Barcelona, Spain) and Lewis wild type and *dwarf* (GH deficient) rats were purchased from Harlan (UK). FSH (Gonal-f) and human choriogonadotropin (hCG; Profasi) were obtained from Serono (Madrid, Spain). Estradiol benzoate (EB) was purchased from Sigma Chemical Co. (St. Louis, MO).

### Tissue dissection

Rats were euthanized by decapitation and trunk blood was extracted. The testis, used for RNA isolation and real-time PCR analyses, were dissected and stored at −80 °C until further processing and assays.

### Experimental design

#### Expression of Lin28/let-7 system and related miRNAs in the testis during postnatal maturation

In **Experiment 1**, the expression profiles of *Lin28a* and *Lin28b* mRNAs, as well as *let-7a, let-7b, mir-9, mir-145* and *mir-132* miRNAs were determined in the testis of rats at different age-points during postnatal maturation: neonatal (PND-1), infantile (PND-15), juvenile (PND-30), early pubertal (PND-38), pubertal (PND-45) and adult (>PND-75) ages, in keeping with previous references^38^; size = 7–8 per group. In addition to RNA analyses by qPCR, *in situ* hybridization (ISH) assays for detection of the cellular distribution of *let-7b* and *mir-145* was applied to adult testicular samples, as described below.

#### Changes in testis expression of the Lin28/let-7 system in models of perturbed puberty

To provide further evidence for the putative roles of this system in the maturational program leading to puberty, a series of expression analyses were conducted in various models of disturbed puberty. In **Experiment 2**, neonatal male rats were exposed to high doses of EB as a model of altered puberty. Alterations of the sex steroid milieu during the critical neonatal period of sexual differentiation are known to disrupt pubertal maturation and gonadotropic function later in life^39^,^40^,^41^. Male rats (n = 10 per group) were injected subcutaneously (sc) on PND-1 with olive oil alone (100 μl; vehicle: control group) or EB (neonatal estrogenization: 500 μg/rat) dissolved in olive oil. On PND-45, the animals were euthanized, trunk blood samples were collected and the testes were dissected out and stored at −80 °C until further processing. In **Experiment 3**, photoperiodic manipulation was used as another model of perturbed puberty. Based on previous evidence showing that either changes in melatonin levels or photoperiod/day length modify the timing of puberty^42^,^43^,^44^, male rats were submitted to constant darkness (CD) during lactation, between PND-10 to -15. Groups of rats reared in standard photoperiod conditions (14-h light/10-h dark) served as controls. Subsets of animals (n = 8–12) were euthanized and testes collected at PND-15 (i.e., immediately after completion of CD) and at puberty (PND-45). Male pubertal maturation was monitored by assessing balano-preputial separation (BPS), during the week before the later age-point. Finally, in **Experiment 4**, a model of perturbed puberty due to postnatal underfeeding during lactation was evaluated. The impact of postnatal under-nourishing on testicular expression of the Lin28*/let-7* system was explored using rats reared in large litters (20 pups/litter), as model of delayed puberty^45^. Rats from normal-sized litters (12 pups/litter) served as controls. After weaning, rats were fed *ad libitum*. Subsets of rats were sacrificed at PND-5, -15 and -45. Male pubertal maturation was assessed by monitoring BPS, as described above.

#### Hormonal regulation of testicular expression of the Lin28/let-7 system and related miRNAs

Regulation of the expression of the *Lin28/let-7* hub was explored *in vivo* in a number of models of neuroendocrine manipulation, known to impact on testicular function. Thus, in **Experiment 5**, regulation of testicular *Lin28/let-7* expression by pituitary gonadotropins was explored. To this end, groups of long-term hypophysectomized (HPX) adult rats (at 4-wk after HPX) received, for 1 week, daily ip injections of hCG (50 IU/24 h, n = 8), FSH (12.5 IU/24 h, n = 7) or a combination of both (n = 8), following previously published protocols^46^,^47^. Animals injected with vehicle (saline) were also included. After the surgery, HPX rats were provided with isotonic saline (0.9%) instead of tap drinking water. Groups of intact animals served as controls. In addition, in **Experiment 6**, testicular *Lin28/let-7* expression levels were monitored in a rat model of GH deficiency, a *dwarf* rat strain derived from the Lewis rat (2–3 months old; Harlan, UK). This model is selectively deficient in GH synthesis with the remaining hormonal parameters within the normal ranges^48^. Finally, in **Experiment 7**, the involvement of other neurohormonal axes in the control of testicular *Lin28/let-7* expression was assessed by measuring changes in mRNA/miRNA levels in models of surgical deprivation of adrenal hormones, by adrenalectomy (ADX), and chemically-induced hypothyroidism by administration of 0.1% aminotriazole in drinking water for three weeks in keeping with previous references^49^,^50^. At the end of all the treatments, the animals were killed by decapitation and testes were processed as described in previous experiments.

### Quantitative real-time PCR

Total RNA was extracted using the Trizol reagent (Invitrogen), and employed for measurement of *Lin28a* and *Lin28b* mRNAs. For assays of miRNA levels, total RNA was extracted with the Ambion® *mir*Vana™ miRNA Isolation Kit (Ambion, Inc; CA, USA). Quality and concentration of RNAs were determined by agarose gel and spectrophotometer ND-1000 NANODROP 385 (Thermo-scientific). Real-time PCR was performed on a BioRad CFX96 Real Time PCR Detection System. For *Lin28a* and *Lin28b* mRNAs, 2 μg of total RNA per tissue sample were treated with RQ1 RNAse-free DNAse-I (Promega) and retro-transcription was carried out in a 30-μl reaction, using AMV reverse transcriptase and random primers (Promega). For PCR, we used SYBR Green qPCR Master Mix (Promega). The primer pairs used were: *Lin28a*-forward: 5′-cccggtggacgtcttt gtg-3′, *Lin28a*-reverse: 5′-cactgcctcaccctccttga-3′; *Lin28b*-forward: 5′-ggatcagatgtggactgtgagaga-3′and *Lin28b*-reverse: 5′-ggaggtagaccgcattctttagc-3′. For data analysis, relative standard curves were constructed from serial dilutions of one reference sample cDNA and the input value of the target gene was standardized to *Hprt* levels in each sample. *Hprt*-forward: 5′-agccgaccggttctgtcat-3′, *Hprt*-reverse: 3′-ggtcataacctggttcatcatcac-5′. PCR was initiated by one cycle of 95 °C for 10 min, followed by 40 cycles of 15 s at 95 °C, 35 s at 60 °C, and 10 s at 72 °C, followed by one hold of 72 °C for 10 min.

For miRNA quantification, cDNA was synthesized by using 10 ng total RNA with TaqMan–specific RT primers and the TaqMan microRNA reverse transcription kit (Applied Biosystems, CA, USA). Thereafter, quantitative RT-PCR was performed using predesigned assays for *Let-7a, Let-7b*, (we studied two representative miRNAs of the *let-7* family belonging to different clusters^13^,^51^), *mir-132, mir-145, mir-9* and *RNU6* (Applied Biosystems). PCR reactions were carried out as follows: 50 °C for 2 min, 95 °C for 10 min, followed by 40 cycles of 95 °C for 15 sec and 60 °C for 1 min. For quantitative miRNA determinations, *RNU6* served as the internal reference.

### MicroRNA *in situ* hybridization

Testes from adult rats were formalin-fixed and paraffin-embedded. Afterwards were sectioned at 10 μm thickness and placed on glass slides. Double digoxigenin (DIG)-labeled miRCURY LNA™ *Let-7b, mir-145* and *RNU6* detection probes (Exiqon, Denmark) were employed in this study. The *in situ* hybridization was performed as described previously^52^.

### Statistical analysis

Expression data were analyzed using Prism GraphPad 5.0 software (GraphPad Software Inc., La Jolla, CA) and were expressed as percentage of the control group in each experiment and presented as mean ± SEM. Statistical significance was determined by *t* Student test (experiments with two groups), one way ANOVA with post hoc Tukey test (experiments with more than two groups and one variable), or two way ANOVA with post hoc Tukey test (experiments with more than two groups and two variables). In addition, Fisher’s exact test was used for the analysis of BPS data (as discontinuous variable). Significance level was set at P ≤ 0.05 and different letters or asterisks indicate statistical significance.

## Results

### Expression of Lin28/let-7 and related miRNAs in the testis during postnatal maturation

The *Lin28/let-7* tandem is subjected to a dual negative feedback regulatory loop. In addition, previous literature and bioinformatic analyses of our group had documented the putative regulatory role in this hub of *mir-145* and *mir-132*, which is highly conserved across most vertebrates. All these factors were included in our expression analyses. Notably, bioinformatic predictions also suggest that *mir-9* might negatively regulate Lin28; yet, this miRNA is more abundantly expressed in the hypothalamus, whereas its expression levels are low in the rat testis (*our unpublished observations*). Hence, *mir-9* expression levels were assayed only in some of our expression analyses, which are presented as Supplemental Fig. 1.

**Figure 1:**
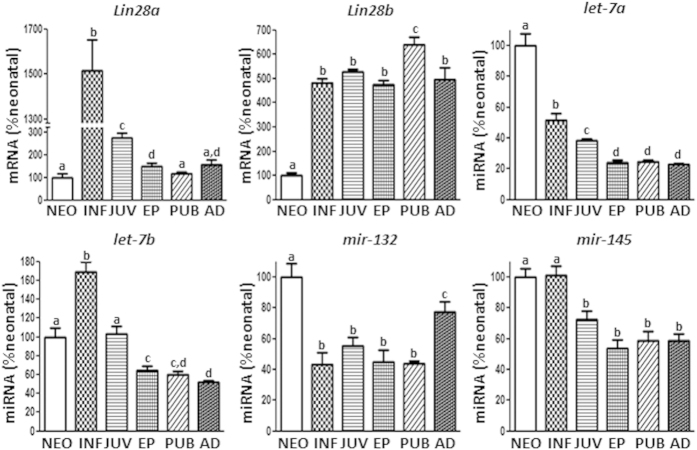
Expression profiles of the components of the *Lin28*/*let-7* axis and related factors in rat testis during postnatal maturation. Expression analyses of *Lin28a* and *Lin28b* mRNAs, as well as *let-7a, let-7b, mir-132*, and *mir-145* miRNAs were conducted in testicular samples from rats at different stages of postnatal development. For presentation of data, the level of expression of mRNAs and miRNAs in neonatal samples was taken as 100%, and the other values were normalized accordingly. Values are expressed as the mean ± SEM. Statistically significant differences between groups are denoted by different superscript letters; groups with different superscript letters are statistically different (*P* < 0.05; ANOVA followed by post hoc Tukey test).

*Lin28a* and *Lin28b* mRNAs displayed low testicular expression during the neonatal period, increasing markedly during infantile period. However, their expression profiles diverged thereafter, so that *Lin28a* mRNA levels showed a subsequent decreased to adulthood, while *Lin28b* mRNA levels remained high from infantile to adulthood, with peak expression around the time of puberty. *Let-7a, let-7b, mir-132*, and *mir-145* (Fig. 1), as well as *mir-9* (Suppl. Figure 1A), showed expression profiles that were grossly opposite to those of *Lin28b*; yet, some slight differences were detected among these miRNAs. All of them declined in expression after the neonatal/infantile period. However, while *let-7a, mir-132* and *mir-9* decreased sharply after PND1, *let-7b* increased between the neonatal and infantile age, to decline thereafter until puberty, whereas *mir-145* levels remained elevated during infantile period and dropped during the juvenile transition. On the other hand, while most of the miRNAs studied remained low at adulthood, *mir-132* expression increased after puberty to reach intermediate levels between neonatal (maximum) and pubertal (minimum) expression. (Fig. 1).

### Pattern of cellular expression of let-7b and mir-145 expression in adult rat testis

**Figure 2:**
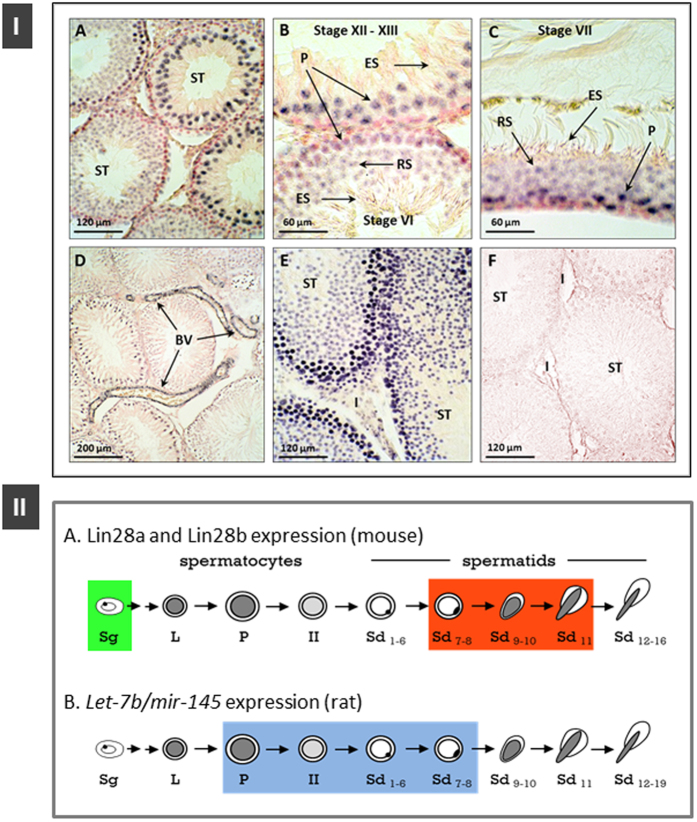
Expression of *let-7b* and *mir-145* in adult rat testis. In the *upper panel*, representative *in situ* hybridization assays of testicular distribution of *let-7b* (**A–C**) and *mir-145* (**D**) in adult rats are shown. ISH signal (purple color) was restricted to germ cells in the seminiferous tubules (***ST***) and was absent in the interstitium (**I**). Germ cells expressing both *let-7b* and *mir-145* corresponded to pachytene spermatocytes (***P*** in **B**,**C**), while a fainter expression was found in round spermatids (***RS***) from stage I to stage VII, and expression was absent in elongating spermatids (***ES***) from stage VIII onwards. A strong expression for *mir-145* was detected in the smooth muscle cells in the interstitial blood vessels (***BV*** in **D**). (**E)** Positive control (hybridized for the ubiquitous microRNA U6); (**F)** Negative control (hybridized with sense probes). In the *lower panel*, schematic representation, comparing the expression of Lin28a (*green box*) and Lin28b (*red box*) protein in mouse germ cells^53^, and of *let-7b* and *mir-145* in rat germ cells (*current study*) is shown. *ST*: seminiferous tubules; *I*: interstitium; *P*: pachytene spermatocytes; *RS*: round spermatids; *ES*: elongating spermatids; *BV*: interstitial blood vessels.

In order to complement our expression data, localization analyses were applied to adult testicular samples to address the pattern of cellular distribution of key elements of the Lin28/*let-7* system. Despite several attempts, following our previously published protocols in mice^53^, our validation tests for detection of Lin28a and Lin28b proteins by immunohistochemistry in rat testis were unsuccessful; these attempts involved the use of different antibodies [anti-Lin28a from Abcam, ref. ab46020; Lin28b-specific polyclonal antibody from ProteinTech Group Inc, ref. 16178-1-AP and Abnova, ref. PAB3154] (Gaytan, Sangiao-Alvarellos, Manfredi-Lozano & Tena-Sempere, *unpublished results*). In contrast, ISH assays successfully detected the expression of two selected miRNAs of the system, namely, *let-7b* and the related miRNA, *mir-145*, in testicular sections from adult rats. Of note, we attempted also to detect the testicular distribution of *mir-132* in rat testis by ISH, but this was unsuccessful probably due to lower endogenous expression levels of this miRNA. Our analyses revealed that *let-7b* miRNA expression in adult rat testis is restricted to germ cells in the seminiferous tubules, in a stage-dependent manner, and is absent in the interstitial areas (Fig. 2, *upper panel, A*). A strong hybridization signal was detected in pachytene spermatocytes (Fig. 2, *upper panel, A–C*), while a fainter signal was observed in round spermatids, from stage I to stage VII. In turn, no expression of *let-7b* was detected by ISH in elongating spermatids (Fig. 2-I, *upper panel, B,C*). The same expression pattern was observed for *mir-145*, with the exception that it was strongly expressed also in smooth muscle cells in the interstitial blood vessels (Fig. 2-I*, upper panel, D*).

### Changes in the profiles of testicular expression of Lin28/let-7 in models of perturbed puberty

**Figure 3:**
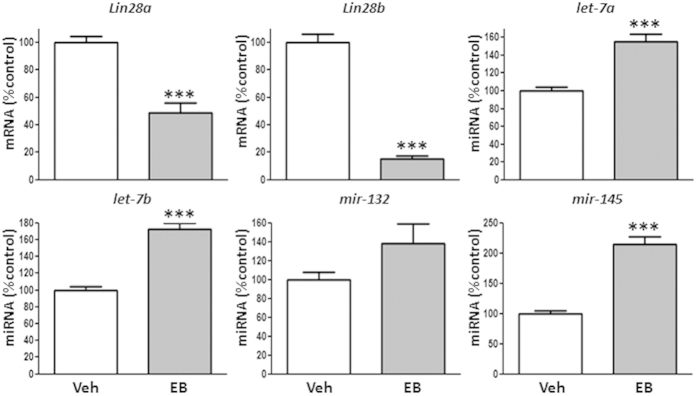
Expression profiles of the components of the *Lin28*/*let-7* axis and related factors in pubertal rat testis rats following neonatal estrogenization. Expression analyses were conducted in testis fragments from rats subjected to a standard protocol of neonatal estrogenization (Estradiol Benzoate, EB); samples were obtained from peripubertal (postnatal day, PND-45) animals. Expression analyses included *Lin28a* and *Lin28b* mRNAs, as well as *let-7a, let-7b, mir-132* and *mir-145* miRNAs. The level of expression of mRNAs and miRNAs in control samples was taken as 100%, and the EB values were normalized accordingly. Values are expressed as the mean ± SEM. *P < 0.05 *vs.* control (Vehicle) group (*t* Student test).

Neonatal administration of EB to male rats perturbed pubertal maturation, as evidenced by decreased serum levels of both gonadotropins, LH and FSH, absent BPS and diminished testicular weights (*data not shown*), in keeping with previous publications^39^,^40^,^41^. This protocol of neonatal estrogenization resulted also in detectable changes in the *Lin28*/*let-7* axis at the expected time of puberty (PND-45). Thus, neonatally estrogenized males displayed decreased *Lin28a* and *Lin28b* mRNA levels. In contrast, *let-7a, let-7b* and *mir-145* miRNA levels (Fig. 3) were significantly higher than in controls, while *mir-132* (Fig. 3) and *mir-9* (Suppl. Figure 1B) expression remained unchanged, although a trend for increased levels was detected for both miRNAs in testes from neonatally estrogenized males.

**Figure 4:**
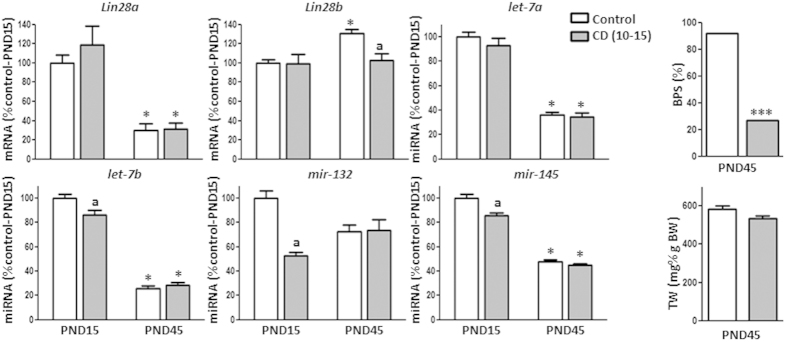
Expression profiles of the components of the *Lin28/let-7* axis and related factors in rat testis following photoperiod manipulation (dark (10–15), constant darkness from postnatal day [PND] 10–15). Studies were conducted at PND-15 and PND-45. For presentation of data, the level of expression of mRNAs and miRNAs at PND-15 and normal photoperiod samples was taken as 100%, and the other values were normalized accordingly. Values are expressed as the mean ± SEM. *P < 0.05 *vs.* PND-15 group for each photoperiodic regimen; (**a**) *P* < 0.05 *vs*. control (normal photoperiod) group for each age (Two-way ANOVA followed by post hoc Tukey test). For BPS analysis, ***P < 0.001 *vs.* CD (10–15) (Fisher’s exact test). BPS: balano-preputial separation, TW: testis weight.

Timed manipulation of photoperiod during postnatal maturation was also used as model of perturbed pubertal timing. Initial analyses, using BPS as external marker of puberty, demonstrated that constant darkness (CD) between PND-10 and -15 consistently delayed the timing of puberty^16^. Thus, at PND-45, <28% of CD males showed complete BPS, as compared with 92% of control animals, reared under standard photoperiod conditions. Analysis of CD males at PND-15 and -45 revealed no changes in testicular *Lin28a* mRNA and *let-7a* miRNA levels, while *Lin28b* expression levels were decreased on PND-45. In contrast, CD males displayed a consistent decline in *let-7b, mir-132* and *mir-145* miRNA abundance at PND-15, but these changes in miRNA levels were transient and were not detected at PND-45, the expected time of puberty (Fig. 4).

**Figure 5:**
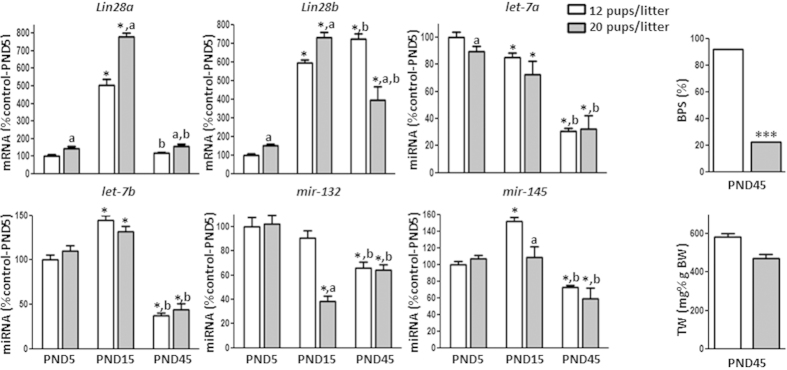
Expression profiles of the components of the *Lin28*/*let-7* axis and related factors in rat testis following postnatal undernutrition, caused by rearing in large litters (20 pups per litter). Analyses were conducted at postnatal day (PND)−5, −15, and −45. The level of expression of mRNAs and miRNAs at PND-5 and 12 pups/litter samples was taken as 100%, and the other values were normalized accordingly. Values are expressed as the mean ± SEM. *P < 0.05 *vs*. PND-5 group for each pups litter group; (**a**) *P* *<* 0.05 *vs.*12 pups/litter for each age; (**b**) *P* *<* 0.05 *vs*. corresponding PND−15 in each pups litter (two-way ANOVA followed by post hoc Tukey test). For BPS analysis, ***P < 0.001 *vs.* control (12 pups/litter) group (Fisher’s exact test). BPS: balano-preputial separation, TW: testis weight.

Finally, testicular expression analyses were also applied to a model of early metabolic distress due to sub-nutrition during lactation, generated by rearing in large litters (LL), which is known to cause delayed puberty^45^. LL males showed an overt delay in the timing of puberty as estimated by the age of BPS in males (at PND-45, only 22% of males reared in large litters had BPS, while 92.6% of control males did). Early postnatal underfeeding resulted in increased *Lin28a* and *Lin28b* mRNA levels at PND-5 and PND-15 (Fig. 5). However, at PND-45, *Lin28b* mRNA levels showed lower abundance compared with control males bred in normal litters, while *Lin28a* mRNA levels remained increased. Changes in testicular miRNA expression were less consistent; thus, while *let-7b* miRNA levels remained unchanged along postnatal maturation, *let-7a* decreased during the neonatal period. Levels of *mir-132* and *mir-145* tended to decrease only at PND-15 in LL males (Fig. 5).

### Hormonal regulation of the Lin28/let-7 system in the testis

**Figure 6:**
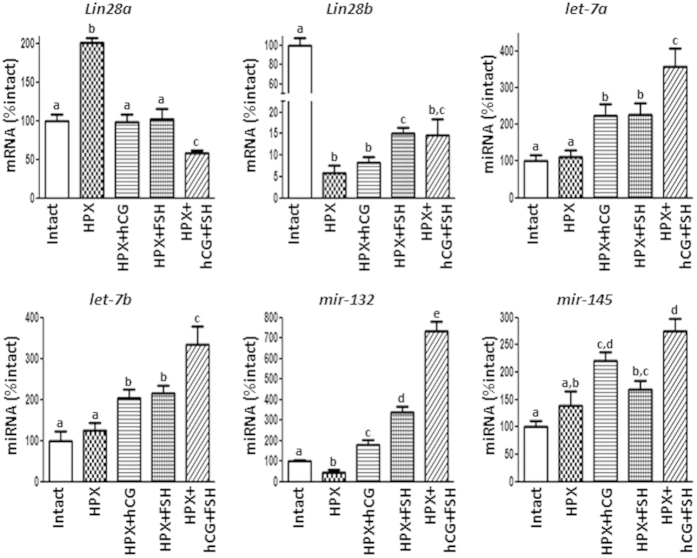
Effects of HPX and gonadotropin replacement on testicular expression *Lin28a* and *Lin28b* mRNAs, as well as *let-7a, let-7b, mir-132,* and *mir-145* miRNAs. The level of expression of mRNAs and miRNAs in control (white bar) samples was taken as 100%, and the other values were normalized accordingly. Values are expressed as the mean ± SEM. Statistically significant differences between groups are denoted by different superscript letters; groups with different superscript letters are statistically different (P < 0.05; ANOVA followed by post hoc Tukey test).

Assessment of hormonal regulation of testicular *Lin28/let-7* expression was first evaluated using HPX rats, with or without gonadotropin replacement, as experimental model. Long-term HPX resulted in the decrease of *Lin28b* mRNA to virtually negligible levels, which were not affected by replacement with hCG, and only modestly but significantly increased by treatment with FSH, alone or in combination with hCG (Fig. 6). In contrast, relative expression levels of *Lin28a* mRNA increased after HPX; a response that was reversed by treatments with hCG or FSH. In fact, administration of both hormones induced lower *Lin28a* levels than those observed in intact rats (Fig. 6). With regard to miRNA expression, HPX did not result in detectable alterations in relative *let-7a, let-7b* or *mir-145* miRNA levels. However, treatment of HPX rats with hCG or FSH alone provoked an increase in their expression levels, which was much more marked when both gonadotropins were administered together (Fig. 6). In contrast, *mir-132* levels diminished after HPX and significantly increased (even over control values) after treatment with hCG or FSH. The combined administration of both gonadotropins caused cumulative effects on *mir-132* miRNA expression (Fig. 6). On the other hand, *mir-9* diminished after HPX, and its levels were recovered only by FSH treatment (Suppl. Figure 1C).

**Figure 7:**
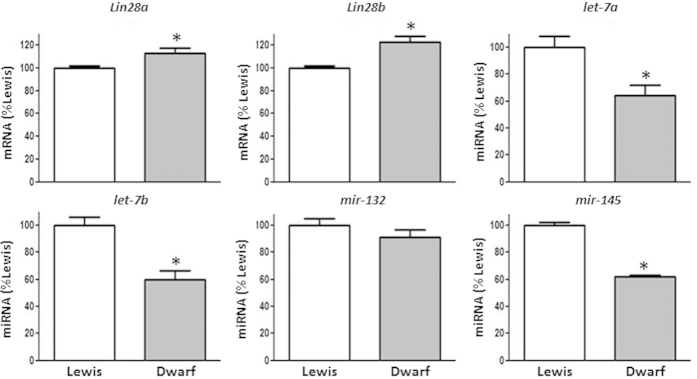
Impact of growth hormone deficiency on the expression profiles of the components of the *Lin28*/*let-7* axis and related factors in adult rat testis. Expression analyses were conducted in testicular samples from wild-type and growth hormone-deficient Lewis rats. For presentation of data, the level of expression of mRNAs and miRNAs in wild-type Lewis rats (white bar) was taken as 100%, and the other values were normalized accordingly. Values are expressed as the mean ± SEM. *P < 0.05 *vs.* control (wild-type Lewis) rats (*t* Student test).

Regulation of testicular expression of the *Lin28/let-7* system by other pituitary hormonal axes was also explored using various approaches. Using a rat model of GH deficiency, we observed that mRNA levels of *Lin28a* and *Lin28b* were significantly higher in *dwarf* rats compared with wild-type Lewis rats. In contrast, *let-7a, let-7b* and *mir-145* miRNA levels were lower in GH-deficient rats compared their controls (Fig. 7). In contrast, assessment of the regulatory roles of adrenal and thyroid hormones, by using models of ADX and chemically-induced hypothyroidism respectively, evidenced that removal of these endocrine signals did not cause significant changes in any of the mRNA and miRNA targets studied (*data not shown*).

## Discussion

In mammals, the three phases of spermatogenesis, namely, mitotic proliferation of spermatogonia, meiosis of spermatocytes, and haploid differentiation of spermatids, progress sequentially to produce male gametes^54^. This is a complex event, regulated by a large number of factors, including miRNAs^30^, which function mainly post-transcriptionally by controlling the stability or translation of their target mRNAs^7^. Among the miRNAs described in rodent testis, the *let-7* family displays prominent expression^53^,^55^. *Let-7* members are negatively regulated by Lin28 proteins^6^. In mammals, two *Lin28* related genes, named *Lin28a* (or *Lin28*) and *Lin28b*, have been described^2^,^3^,^4^. *Lin28* paralogs are highly conserved across evolution^5^, therefore suggesting relevant, presumably overlapping regulatory functions. However, unequivocal demonstration of their relative roles has remained elusive. Recent works suggest a role for Lin28/*let-7* axis in fertility and spermatogenesis. Yet, our knowledge of the patterns of expression, as well as the hormonal and developmental regulation, of the elements of this system in the testis is incomplete.

Our present data extend and complete previous studies from our group in the mouse testis that documented that Lin28a and Lin28b display totally different expression patterns of distribution, there-fore suggesting different roles in the regulation of male gametogenesis and gonadal function during postnatal maturation in rodents. Thus, while Lin28a was restricted to undifferentiated and type A spermatogonia, Lin28b protein was detected in spermatids and Leydig cells^53^. Expression of Lin28a in undifferentiated and early differentiating type A spermatogonia has been independently confirmed in the mouse^33^,^37^. Notably, comparison of expression analyses of *Lin28a/Lin28b* transcripts (and *let-7* miRNAs) in the testis of rats (*present results*) and mice^53^ reveals a strikingly similar profile between these two rodent species, suggesting a notable degree of conservation of the Lin28 system in the testis. This is especially relevant for the present work, since our attempts to map the cellular distribution of Lin28a and Lin28b proteins in the rat testis failed, despite successful immunohistochemical characterization in the mouse testis^53^, probably due to species-differences in the specificity and sensitivity of the antibodies commercially available.

Nonetheless, on the basis of the above similarities, we consider tenable that Lin28a and Lin28b share similar distribution in the testis of rats and mice. This would imply that Lin28a protein is expressed in undifferentiated and type-A spermatogonia in the adult rat testis, whereas Lin28b protein in spermatids and Leydig cells. Such distribution fits well with the pattern of cellular expression of two selected miRNAs, known to repress Lin28a/Lin28b, namely, *let-7b* and *mir-145*, as detected by ISH adult rat testis. Thus, *let-7b* was strongly expressed in pachytene spermatocytes, which are negative for Lin28a or Lin28b, and progressively declined at later stages of spermatogenesis, with weaker signal in round spermatids from stage I to stage VII and absence of expression in elongating spermatids, which display high expression levels of Lin28b protein in the mouse. The same expression pattern was detected for *mir-145*, with the exception that it was strongly expressed also in smooth muscle cells in the interstitial blood vessels, in accordance with previous works^56^,^57^; *mir-145* is known to indirectly suppress *Lin28a/Lin28b* expression by repressing its transcriptional activator, c-Myc^11^,^12^. All in all, the above expression data from two rodent species strongly suggest a dynamic reciprocal regulation of *Lin28a* and *let-7* (and related miRNAs) along the spermatogenic cycle, whereby high expression of *Lin28a* or *Lin28b* is associated with (and possibility caused by) low or absent expression of regulatory miRNAs in specific cell types of the seminiferous epithelium; this profile of reciprocal changes is depicted in Fig. 2-II. Admittedly, however, we do not exclude the possibility that some of the reported changes of these mRNA and miRNA species might stem from testicular cell types other than germ cells; this might well be the case for *Lin28b*, whose protein has been detected in mouse Leydig cells^53^, as well as *mir-145*, which is highly expressed in smooth muscle cells of interstitial blood vessels of rat testis (*present results*).

As mentioned above, compelling biochemical data suggest the existence of a double negative feedback loop whereby Lin28a/Lin28b redundantly represses the synthesis of mature *let-7* miRNAs, which in turn suppress Lin28 levels. In good agreement, an inverse correlation between the relative expression levels of *Lin28* transcripts and their negative miRNA regulators was found in different conditions. For instance, during neonatal period, *Lin28a/Lin28b* mRNA expression was minimum and (especially for *Lin28b*) increased thereafter, whereas *let-7* and also *mir-132, mir-9* and *mir-145* miRNAs abundance was maximal on PND1, decreasing progressively along postnatal maturation. The same profiles of inverse relationship was found in models of altered puberty due to neonatal estrogenization, where *Lin28a/Lin28b* mRNA levels were consistently reduced while *let-7* levels were increased, and in the dwarf GH-deficient rat model, which displayed opposite profiles. The same applies to the dynamic changes reported in the seminiferous tubules, with absent expression of *let-7b* and *mir-145* in cell types of the seminiferous epithelium with high expression of Lin28a (spermatogonia) or Lin28b (round/elongating spermatids) in the mouse. Admittedly, such inverse relationship was not always detected in other models of pubertal or hormonal manipulation. This partial lack of inverse correlation might reflect differences in the cellular distribution of the different mRNAs and miRNAs under analysis, which due to methodological limitations could not documented for all the targets in the rat testis. Likewise, we cannot rule out that such partial mismatch in inverse correlations, and even part of the net expression changes reported here, may derive from the profound changes in testicular cellularity of the testis following some of the above experimental manipulations, which differentially affect the various testicular cell populations and might mask subtler, cell-specific regulatory events.

Different models of perturbed puberty and hormonal manipulation, targeting key endocrine axes with proven roles in the control of testicular function, were explored as a means to provide indirect evidence for the relevance of the Lin28*/let-7* system in the regulation of the rat testis and its modulation by key developmental and hormonal signals. Analyses in models of perturbed puberty, with variable impact on pubertal timing, revealed that early manipulations of the hormonal and nutritional milieu, as well as photic cues, could influence the profiles of expression of different members of the *Lin28/let-7* hub along postnatal testicular maturation. This impact, however, varied amply among the experimental manipulations, with a marked alteration of the system in the model of neonatal estrogenization, and less consistent changes in models of delayed puberty caused by postnatal photoperiodic manipulation or subnutrition. Admittedly, this might reflect the differential effects of such experimental manipulations on the cellularity of the testis. Thus, neonatally estrogenized rats, in which puberty was altered, showed decreased testicular *Lin28a* and *Lin28b* mRNA levels, while *let-7a, let-7b* and *mir-145* miRNAs expression levels were enhanced. In this model, clear alterations in the cellular composition of the testes are found, including spermatogenic arrest with degenerating germ cells, and delayed maturation of Sertoli and Leydig cells, which might partially explain the reported changes in the above targets. However, while this phenomenon might explain loss of expression of some transcripts (e.g., *Lin28b*), the arrest of spermatogenesis at early stages can hardly justify the observed increases in miRNAs, such as *let-7b* and *mir-145*, which are abundantly expressed in spermatocytes and early spermatids, therefore suggesting additional regulatory phenomena reciprocally linking *Lin28* and *let-7* expression in the testis.

Interestingly, recent studies from our group assessing the dynamics in the hypothalamic expression of the *Lin28/let-7* system documented that, as is the case for the testis, at central levels reciprocal changes between *Lin28* and *let-7* expression levels are detectable along postnatal maturation and in neonatally estrogenized rats. However, the trends of such changes were diametrically opposite between the hypothalamus and the testis, so that the hypothalamic *Lin28/let-7* ratio decreased during maturation and increased after neonatal estrogenization^16^, whereas the contrary applies to the testis. These findings suggest that the bidirectional regulatory loops between *Lin28* and *let-7* miRNAs might operate at different levels of the reproductive axis, but the nature and functional implications of this regulatory loop likely vary at different tissues.

Testicular expression of the elements of the *Lin28/let-7* system was also affected in models of photoperiodic and nutritional manipulation, as well as after HPX, suggesting the convergence of multiple regulatory signals. Replacement studies in HPX rats unveiled that while *Lin28a* mRNA levels increased after pituitary hormone removal and decreased after gonadotropin replacement, FSH and/or hCG (as agonist of LH) up-regulated to a variable extent *Lin28b* and miRNA levels. However, whereas *Lin28b* mRNA was only marginally increased by FSH, the relative expression levels of *let-7a, let-7b* and *mir-132* were more robustly increased by FSH and hCG, or their combination, which suggests a strong gonadotropic regulation that may take place at the tubular and/or interstitial compartment of the testis. The dissociation of *Lin28a* and *Lin28b* expression reported here is reminiscent of the observed changes in the testicular expression of both targets a model of hypogonadotropic hypogonadism in mice^16^. Alike, the differential responses of the different targets might reflect the variable impact of gonadotropins on the different cell populations of the testis.

In addition to pituitary gonadotropins, different endocrine signals regulate testicular function. Among them, significant biological actions of glucocorticoids, thyroid hormones and GH^58^,^59^ have been demonstrated in rodent testis. For this reason, we studied testicular expression of *Lin28a* and *Lin28b* mRNAs and *let-7a, let-7b, mir-145* and *mir-132* miRNAs in models of GH deficiency, hypothyroidism and in adrenalectomized rats. Only conditions of GH withdrawal were associated with consistent, reciprocal changes of *Lin28/let-7* (and related miRNA) levels, therefore suggesting a negligible role of thyroid and adrenal hormones in the control of this system in the testis, and the putative involvement of changes in the *Lin28/let-7* system in the generation of testicular alterations due to GH deficiency^58^,^59^,^60^. Again, changes in testicular cellularity might partially explain some of the findings, although they are difficult to reconcile with some of the data (e.g., increased *Lin28a* and *Lin28b* levels in dwarf rats). Similarly, since our GH-deficient model was congenital, while the other hormonal models were induced at adulthood, it remains possible that the impact was greater in the former (dwarf rats) than in the latter.

Admittedly, a limitation of our study is that, while testicular expression profiles are defined in a large number of developmental stages and experimental conditions, it falls short in providing mechanistic information. Nonetheless, the present data are valuable to further explain and provide the basis for previous findings coming from functional genomic studies. Thus, both congenital elimination of Lin28a^36^ and embryonic over-expression of *let-7*^24^ have been shown to induce a reduction of the germ cell pool, with Lin28a KO mice displaying reduced fertility in adulthood^36^. Moreover, germ cell-specific knockout of Lin28a has been shown to reduce testis weight and sperm number, and to impair spermatogonial cell proliferation^37^. These findings, however, stem from congenital models, which are likely endowed with profound developmental defects and, hence, might be more difficult to translate to pubertal or adult physiology. Our current findings complement those previous observations and help to provide educated hypotheses in the testicular roles of the Lin28/let-7 system in the postnatal testis in normal and pathophysiological conditions. For instance, on the basis of previous findings and our current data, it is arguable that the concomitant increase in *Lin28a/Lin28b* and decrease of *let-7* expression during post-natal maturation might favour completion of spermatogenesis during puberty, while reversion of the *Lin28/let-7* ratio might contribute to perturbation of spermatogenesis in models such as neonatal estrogenization. While additional mechanistic studies are needed to fully support these hypotheses, our present results, which characterize the profiles of developmental expression and hormonal regulation of the *Lin28/let-7* system in the rodent testis, help to consolidate the view that the elements of this system are involved in the dynamic control of male gonadal maturation and function in mammals.

## Additional Information

**How to cite this article**: Sangiao-Alvarellos, S. *et al.* Testicular expression of the *Lin28/let-7* system: Hormonal regulation and changes during postnatal maturation and after manipulations of puberty. *Sci. Rep.* **5**, 15683; doi: 10.1038/srep15683 (2015).

## Supplementary Material

Supplementary Information
