# Supplementary material for: Testicular expression of the Lin28/let-7 system: Hormonal regulation and changes during postnatal maturation and after manipulations of puberty
Source: Sci Rep. 2015 Oct 23;5:15683. doi: 10.1038/srep15683 (PMC4616161; doi:10.1038/srep15683)

SREP-15-17612-v2 (*Revised*)

SUPPLEMENTAL MATERIAL

Testicular expression of the *Lin28/let-7* system: Hormonal regulation and changes during postnatal maturation and after manipulations of puberty

Sangiao-Alvarellos S[1,3*#], Manfredi-Lozano M[1#], Ruiz-Pino F[1], León S[1], Morales C[2], Cordido F[3], Gaytán, F[1], Pinilla L[1] and Tena-Sempere M[1,4*]

[1]Department of Cell Biology, Physiology and Immunology, University of Córdoba; CIBER Fisiopatología de la Obesidad y Nutrición, Instituto de Salud Carlos III; and Instituto Maimónides de Investigación Biomédica (IMIBIC)/Hospital Universitario Reina Sofía, 14004 Córdoba, Spain; [2]Department of Pathology, University of Córdoba, 14004 Córdoba, Spain; [3]Department of Medicine, School of Health Science, University of A Coruña, and Instituto de Investigación Biomédica de A Coruña (INIBIC), 15006, A Coruña, Spain; and [4]FiDiPro Program, Department of Physiology, University of Turku, Kiinamyllynkatu 10, FIN-20520 Turku, Finland

Legend to Supplemental Figures

Supplemental Figure 1: *Profiles of expression of mir-9 in the rat testis during postnatal maturation and in models of perturbed puberty and/or gonadal function.* In **panel A**, the expression profile of *mir-9* in rat testis during postnatal maturation is presented. Expression analyses were conducted in testicular samples from rats at different stages of postnatal development: Neonatal (NEO), Infantile (INF), Early Pubertal (EP), Pubertal (P) and Adult (AS). For presentation of data, the level of expression in neonatal samples was taken as 100%, and the other values were normalized accordingly. In **panel B**, the expression profile of *mir-9* in pubertal rat testis rats following neonatal estrogenization is shown. Expression analyses were conducted in testicular samples from rats subjected to a standard protocol of neonatal estrogenization (Estradiol Benzoate, EB); samples were obtained from peripubertal (postnatal day, PND-45) animals. Male rats neonatally treated with vehicle (oil, VEH) served as controls. The level of expression of *mir-9* in control (Veh) samples was taken as 100%, and the EB values were normalized accordingly. In **panel C**, the effects of HPX and gonadotropin replacement on testicular expression of *mir-9* are displayed. The level of expression of *mir-9* in intact (white bar) samples was taken as 100%, and the other values were normalized accordingly. Values are expressed as the mean ± SEM. Groups with different superscript letters are statistically different ($P < 0.05$; ANOVA followed by post hoc Tukey test).

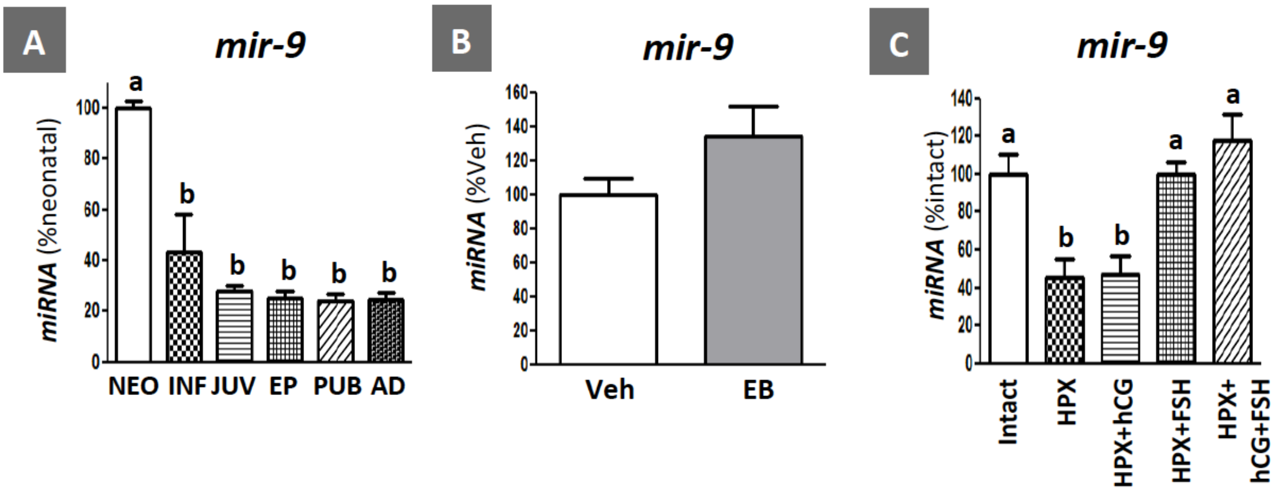

Supplement: Supplementary Information [file srep15683-s1.pdf]
